# LncRNA SNHG7 promotes the proliferation of nasopharyngeal carcinoma by miR-514a-5p/ELAVL1 axis

**DOI:** 10.1186/s12885-020-06775-8

**Published:** 2020-05-05

**Authors:** Weiqun Hu, Haolin Li, Shaozhong Wang

**Affiliations:** 1 Department of Otolaryngology, Putian University Affiliated Hospital, Putian, 351100 Fujian China; 2 Department of Otolaryngology, Xinxiang First People’s Hospital, Xinxiang, 453000 Henan China; 3 Otolaryngngology of Qinghai Provincial People’s Hospital, Gonghe Road No.2, Xining, 810007 Qinghai Province China

**Keywords:** SNHG7, miR-514a-5p, ELAVL1, Nasopharyngeal carcinoma

## Abstract

**Background:**

Nasopharyngeal carcinoma (NPC), with distinct geographical distribution, has gathered public attention. Despite that radiotherapy and chemotherapy are applied to treat NPC, cell metastasis still cannot be avoided. Numerous works have elucidated that lncRNAs are essential players in the development of multiple cancers. LncRNA SNHG7 has been reported as a contributing factor in the occurrence of certain cancers, but its mechanism in NPC deserves further investigation. The purpose of the study is to figure out the role and molecular regulation mechanism of SNHG7 in NPC.

**Methods:**

The role of SNHG7 in NPC was verified by CCK-8, colony formation, EdU staining, western blot and capase-3 assays. The interactions between SNHG7/ELAVL1 and miR-514a-5p were confirmed by RNA pull down, RT-qPCR, RIP and luciferase reporter assays.

**Results:**

SNHG7 was upregulated in NPC cells, and absence of SNHG7 suppressed cell proliferation as well as promoted cell apoptosis in NPC. Furthermore, SNHG7 was confirmed to bind with miR-514a-5p and negatively modulate miR-514a-5p expression. Besides, miR-514a-5p was found to be able to bind with ELAVL1 and negatively regulate ELAVL1 mRNA and protein expressions. In the end, rescue assays demonstrated that the miR-514a-5p deficiency restored the NPC progression inhibited by SNHG7 silence, and ELAVL1 partly counteracted the restoration caused by miR-514a-5p inhibitor in HNE1 cells.

**Conclusions:**

LncRNA SNHG7 promotes the proliferation and migration of nasopharyngeal carcinoma by miR-514a-5p/ ELAVL1 axis.

## Background

Nasopharyngeal carcinoma (NPC) is one of the malignancies that can easily invade to adjacent regions and pose a serious threat to people’s health all over the world [1, 2]. Although radiotherapy and chemotherapy are adopted to treat NPC patients, the prognosis of NPC remains disappointing [3, 4]. Meanwhile, the improvement of early diagnosis is also a problem difficult to solve [5]. Therefore, it is essential to explore the molecular mechanism underlying NPC malignant behaviors such as proliferation and metastasis.

Long non-coding RNAs (LncRNAs) are composed of more than 200 nucleotides in terms of length, and are related to diverse important biological processes [6, 7]. Importantly, a growing number of evidences suggested that lncRNAs play a part in the tumorigenesis of various cancers and regulate the tumor progression [8, 9]. For example, lncRNA LINC01503 promotes proliferation and invasion of colorectal cancer cells via mediating miR-4492/FOXK1 axis [10]. LncRNA AGAP2-AS1 functions as an oncogene in glioblastoma multiforme and leads to a poor prognosis [11]. Nonetheless, lncRNA LOC285194 serves as a tumor-inhibitor in non-small cell lung cancer through targeting p53 [12]. Meanwhile, a wide variety of lncRNAs have been reported to be regulators in NPC [13]. For instance, lncRNA CASC2 regulates the proliferation and apoptosis of NPC through targeting miR-18a-5p/RBBP8 axis [14]. LncRNA ANCR promotes cell proliferation and radioresistance by repressing PTEN expression in NPC [15]. LncRNA ARHGAP42 promotes the migration and invasion of NPC cells via PI3K/Akt signaling pathway [16]. Recent studies have revealed that lncRNA small nucleolar RNA host gene (SNHG7) plays an oncogenic role in numerous cancers, such as bladder cancer [17], colorectal cancer [18], esophageal cancer [19] and gastric cancer [20]. A recent study by Wang et al. showed that SNHG7 was highly expressed in NPC tissues and knockdown of SNHG7 inhibited proliferation, migration and invasion in NPC cells via inhibiting ROCK1 [21]. However, the role and molecular mechanism of SNHG7 in NPC need to be further explored.

In this discovery, we attempt to further investigate the biological function and regulatory mechanism of SNHG7 in NPC. It was identified that lncRNA SNHG7 promotes the proliferation and migration of NPC by miR-514a-5p/ELAVL1 axis, which provided new thoughts for the improvement of molecular-targeted treatment for NPC.

## Methods

### Cell lines and culture

CNE1, CNE2, C666–1 and HNE1, the four acknowledged NPC cell lines and a nasopharyngeal epithelial cell line (NP69) were incubated in RPMI-1640 medium (Gibco, Grand Island, NY, USA) containing 10% fetal bovine serum (Gibco). The catalogue for these cell lines and the commercial company are presented as below: CNE1 (Shanghai Biological Technology Co., Ltd. Enzyme research, China, ml053103); CNE2 (Shanghai Biological Technology Co., Ltd. Enzyme research, Shanghai, China, ml053100); C666–1 (Shanghai Biological Technology Co., Ltd. Enzyme research, Shanghai, China, ml055596); HNE1 (Otwo BioTech, Shenzhen, China, HTX2639); NP69 (Shanghai Biological Technology Co., Ltd. Enzyme research, Shanghai, China, ml056672). All cell lines were acquired commercially from the Cell Resource Center of Shanghai Institutes for Biological Sciences (China) and maintained in a 5% CO_2_ and 37 °C incubator. Once adherent growth, cells were digested with trypsin. Cells in logarithmic growth phase were collected for further analysis.

### Quantitative real-time polymerase chain reaction (qRT-PCR)

The total RNA in C666–1 and HNE1 cells was isolated in strict accordance with TRIzol reagent (Invitrogen, Carlsbad, CA, USA). Isolated RNAs were reversely transcribed into complementary deoxyribose nucleic acid (cDNA) using Fast Quant RT Kit (TaKaRa, Otsu, Shiga, Japan). The cDNA template was used for qRT-PCR. LightCycler® 480 SYBR Green I Master (Roche, OR, USA) was used in the Roche LightCycler® 480 System. All experimental results were calculated and presented as 2^-ΔΔCt^, with the normalization of GAPDH and U6.

### Cell transfection and plasmids

The sh-SNHG7#1/#2 (#1: 5′-GGAAGATGTTTGTCGGCATCT-3′; and #2: 5′-GCCTGGGTGTTGCTGTGTATT-3′) and sh-ELAVL1, together with the negative control shRNA (sh-NC) (5′-TGAGACGAAGCTTCGTCTCGT-3′), were obtained from Genepharma (Shanghai, China) and transfected into C666–1 or HNE1 cells as per the protocol of Lipofectamine 2000 (Invitrogen, USA). MiR-514a-5p mimics, NC mimics, the oligonucleotide against miR-514a-5p (miR-514a-5p inhibitor) and control oligonucleotide (NC inhibitor) were designed and synthesized at Genepharma (Shanghai, China). After cells were grown to 50–80%, cells were transfected with the aforementioned plasmids or their controls, respectively.

### Cell counting kit-8 (CCK-8) assay

Forty-eight hours after transfection, C666–1 or HNE1 cells were harvested with trypsin and placed into 96-well plates with 1000 cells in each well. The cell proliferation was tested via examining the absorbance at 450 nm after re-seeding cells for 0, 24, 48, 72 and 96 h with CCK-8 (Dojindo, Kumamoto, Japan) in light of the specifications.

### Colony formation assay

The transfected C666–1 or HNE1 cells were trypsinized into single cells and planted in 96-well plate for 2 weeks with 5% CO_2_ and 100% humidity without changing the medium at 37 °C. 1% formaldehyde solution and 0.1% crystal violet were separately used to fix the washed cells for 10 min and stain them for 15 min. Single-cell colony formation ability was assessed via counting the colonies (no less than 50 cells) number.

### EdU staining

4 × 10^4^ C666–1 or HNE1 cells were transfected on the basis of experiment design and put in 24-well plates on sterile coverslips. EdU assay kit from RiboBio (Guangzhou, Guangdong, China) was utilized for evaluating cell proliferation based on the user guide. Cell nuclei were subjected to 4–6-diamidino-2-phenylindole (DAPI; Beyotime, Shanghai, China) staining and taken as the positive control. Images of stained cells were acquired with Laser confocal microscopy (FV300, Olympus, Tokyo, Japan).

### Western blot

Transfected C666–1 or HNE1 cells were rinsed twice in pre-chilled PBS solution on ice and cultivated in radioimmunoprecipitation assay (RIPA; Beyotime). Following separation on 15% SDS-PAGE, protein samples were transferred onto PVDF membranes (Millipore, Billerica, MA, USA). 5% skim milk powder solution was added for blocking non-specific binding. The rinsed membranes were cultured with primary antibodies at 4 °C all night and then with the corresponding secondary antibodies (Abcam, Cambridge, MA, USA) at room temperature for 2 h. Thereafter, the enhanced chemiluminescence Kit (ECL; Millipore) was applied for detection of immunoreactive bands.

### Measurement of caspase-3 activity

Caspase-3 activity was utilized to measure cell apoptosis using the Caspase-3 Activity Kit (Solarbio, Beijing, China). The total proteins from transfected C666–1 or HNE1 cells were reaped and added into the 96-well plates containing assay buffer and caspase-3 substrate, followed by 4 h of culturing. Measurement of caspase-3 activity was conducted by a microplate reader at 405 nm.

### Subcellular fractionation assay

PARIS™ kit from Ambion (Austin, TX, USA) was used to separate nucleus and cytoplasm of C666–1 or HNE1 cells. The cultured cells were harvested and washed on ice in PBS, following culturing in 500 μl of pre-cooled cell fractionation buffer. The supernatant (cytoplasm) was isolated from centrifuged cells. The final nuclear and cytoplasmic RNA was reverse-transcribed and subjected to qRT-PCR to test the SNHG7 expression in nucleus and cytoplasm.

### RNA pull-down assay

For RNA pull-down assay, the RNA-protein mixture was obtained via culturing 2 μg of biotinylated probes with cell extracts at 30 °C for 30 min. Following treatment with Streptavidin Sepharose at room temperature for 1 h, the complex was pulled down. The bound RNAs were extracted, purified, and analyzed with qRT-PCR.

### RNA Immunoprecipitation (RIP) assay

The Magna RIP™ RNA-Binding Protein Immunoprecipitation Kit (Millipore, USA) was employed to implement RIP assay in C666–1 or HNE1 cells. Cultured cells from RIP lysis buffer were collected and cultivated with beads and antibody of interest with rotation for 30 min. Normal Rabbit IgG was seen as control. After digestion, precipitated RNAs were isolated and tested by qRT-PCR.

### Luciferase reporter assay

The synthetic wild-type and mutated binding sites of miR-514a-5p in SNHG7 or ELAVL1 sequences were inserted into the pmirGLO vector (Promega, Madison, WI, USA). The reporter plasmids SNHG7-WT/Mut and ELAVL1–3′-UTR-WT/Mut were generated. C666–1 and HNE1 cells were co-transfected with 50 nM of miR-514a-5p mimics or NC mimics and 500 ng/mL reporter plasmids for 48 h. The final luciferase intensity was examined by the Dual-Glo® Luciferase Assay System (Promega).

### Statistical analysis

Statistical Product and Service Solutions (SPSS) 17.0 software (SPSS Inc., Chicago, IL, USA) was applied for statistical analysis. Biological triplicate was required for each experiment. Quantitative data were exhibited as mean ± SD. Difference comparison between groups were analyzed with independent t-test or ANOVA. In all analyses, *p* < 0.05 was seen as the significant level.

## Results

### SNHG7 is highly expressed and knockdown of it represses cell proliferation and facilitates cell apoptosis in NPC

**Fig. 1:**
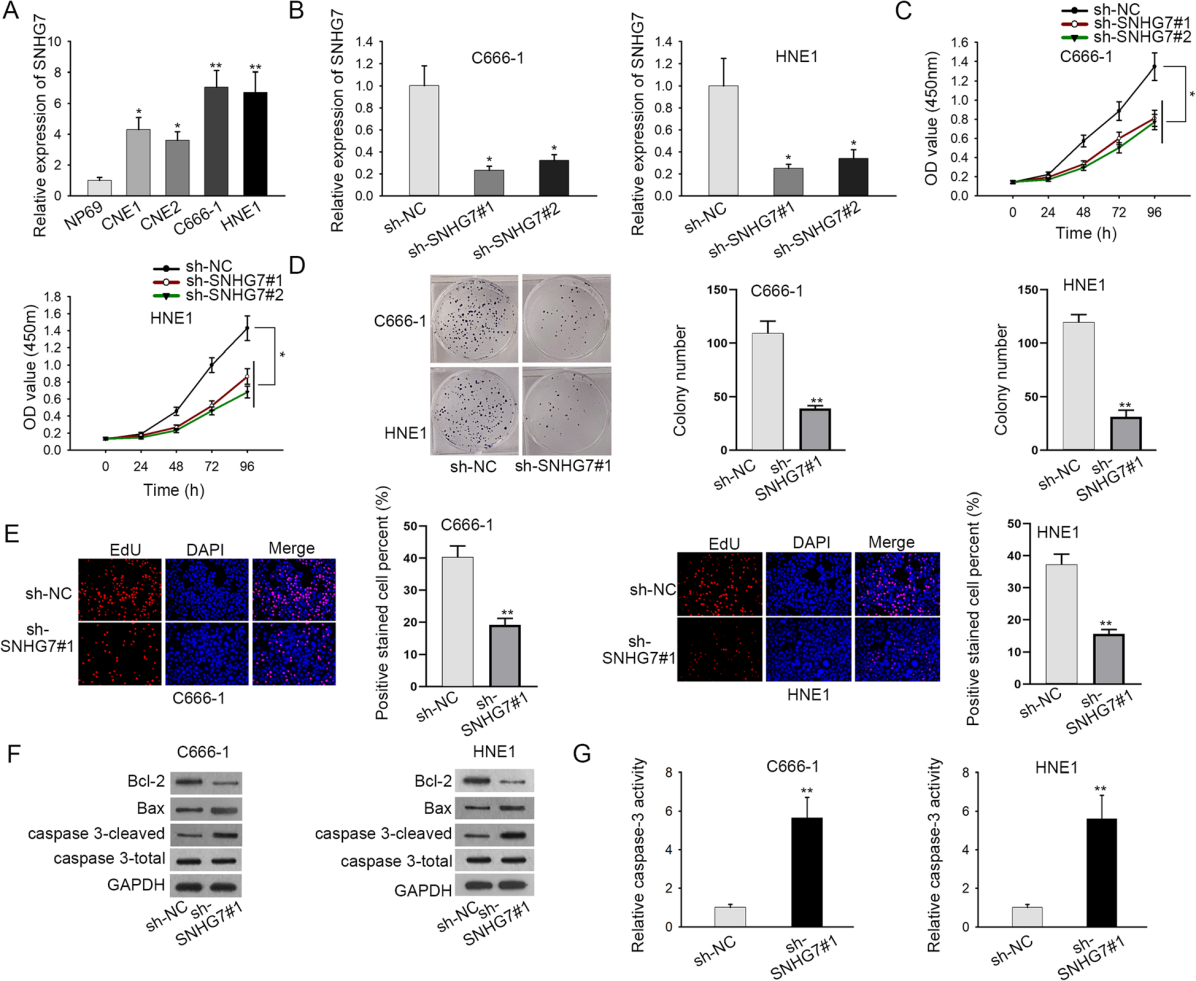
SNHG7 is highly expressed and knockdown of it represses cell proliferation and facilitates cell apoptosis in NPC. **a** The expression of SNHG7 in NPC cells and nasopharyngeal epithelial cell line was examined by RT-qPCR. **b** The knockdown efficiency of SNHG7 was detected via RT-qPCR. **c**-**e** The effect of SNHG7 deficiency on cell growth in NPC was measured by CCK-8, colony formation and EdU assays. **f** Western blot assay examined the expression of apoptosis-related proteins in transfected cells. These blots were cropped. The uncropped blots were shown in Supplementary figure 2. **g** Caspase-3 activity reflected cell apoptosis in transfected cells. GAPDH was used as an internal control. Error bars represent the mean ± SD of at least three independent experiments. ^*^P < 0.05, ^**^P < 0.01

First, the expression status of SNHG7 in NPC cells was explored. As displayed in Fig. 1a, the expression of SNHG7 was notably upregulated in NPC cell lines (CNE1, CNE2, C666–1 and HNE1) in comparison with normal human nasopharyngeal epithelium NP69. The knockdown efficiency of SNHG7 was evaluated by RT-qPCR analysis after C666–1 and HNE1 cells were transfected with sh-SNHG7#1/2 or sh-NC. An obvious downregulation of SNHG7 was observed in sh-SNHG7#1/2-transfected cells (Fig. 1b). According to CCK-8, colony formation and EdU assays, SNHG7 knockdown significantly inhibited cell proliferation ability in C666–1 and HNE1 cells (Fig. 1c-e). Moreover, western blot assay revealed that sh-SNHG7#1 increased the expression of pro-apoptosis proteins (Bax and caspase3-cleaved), and decreased the expression of anti-apoptosis protein (Bcl-2) in C666–1 and HNE1 cells (Fig. 1f, S1A and S2A). Furthermore, the caspase-3 activity was enhanced by knocking down SNHG7 in C666–1 and HNE1 cells (Fig. 1g). Taken together, SNHG7 is highly expressed in NPC cells and knockdown of it represses cell proliferation and facilitates cell apoptosis of in NPC.

### SNHG7 sponges miR-514a-5p in NPC

**Fig. 2:**
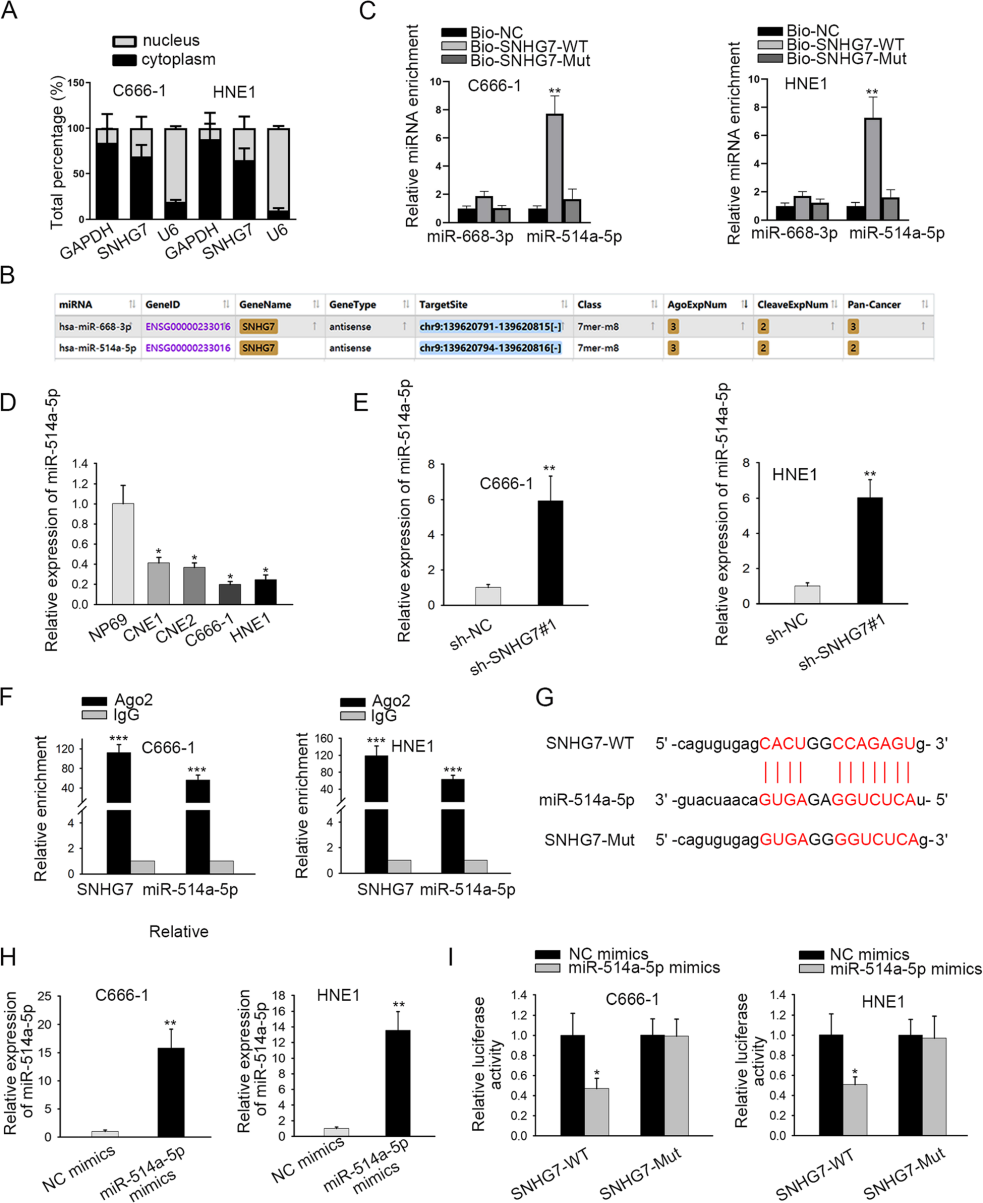
SNHG7 sponges miR-514a-5p in NPC. **a** Subcellular fractionation assay detected the distribution of SNHG7 in C666–1 and HNE1 cells. **b** starBase website screened out two miRNAs that possess the highest binding ability with SNHG7. **c** RNA pull down assay analyzed the interaction between SNHG7 and miR-668-3p (or miR-514a-5p). **d** The expression of miR-514a-5p in NPC cells and nasopharyngeal epithelial cell line was quantified by RT-qPCR. **e** The influences of SNHG7 silencing on miR-514a-5p expression were evaluated via RT-qPCR. **f** RIP assay proved that SNHG7 and miR-514a-5p coexisted in RISC. **g** The potential binding sites between SNHG7 and miR-514a-5p were displayed. **h** The overexpression efficiency of miR-514a-5p was quantified by RT-qPCR. **i** Luciferase activity assay demonstrated that SNHG7 could bind with miR-514a-5p. GAPDH or U6 was used as an internal control. Error bars represent the mean ± SD of at least three independent experiments. ^*^P < 0.05, ^**^P < 0.01, ^***^P < 0.001

After understanding the biological role of SNHG7, we set out to probe the molecular mechanism of SNHG7 in NPC. At first, the distribution of SNHG7 in nucleus and cytoplasm was detected by subcellular fractionation assay. Results depicted that SNHG7 was mainly localized in cytoplasm (Fig. 2a). LncRNAs can serve as ceRNAs to sponge miRNAs and regulate downstream genes in cytoplasm [22]. By browsing Starbase, 2 miRNAs (miR-668-3p and miR-514a-5p) were found to potentially bind with SNHG7 (Fig. 2b). Besides, RNA pull-down assay depicted that miR-514a-5p was remarkably enriched in Bio-SNHG7-WT instead of Bio-NC or Bio-SNHG7-Mut group (Fig. 2c). Hence, we performed RT-qPCR so as to figure out the expression level of miR-514a-5p in NPC cells. As shown in Fig. 2d, miR-514a-5p expression was evidently downregulated in NPC cell lines in comparison with normal human nasopharyngeal epithelium. Moreover, sh-SNHG7#1 dramatically increased the expression of miR-514a-5p in C666–1 and HNE1 cells (Fig. 2e). Afterwards, RIP assay demonstrated that SNHG7 and miR-514a-5p were enriched in anti-Ago2 group rather than anti-IgG group (Fig. 2f). The miR-514a-5p binding sites on SNHG7 were identified starBase and presented in Fig. 2g. Next, RT-qPCR analysis displayed that the expression of miR-514a-5p was increased by miR-514a-5p mimics in C666–1 and HNE1 cells (Fig. 2h). Moreover, the luciferase activity of pmirGLO-SNHG7-WT was reduced by miR-514a-5p mimics while the luciferase activity of pmirGLO-SNHG7-Mut showed no pronounced change in different groups (Fig. 2i). Overall, SNHG7 acts as a sponge for miR-514a-5p in NPC.

### ELAVL1 is a downstream target gene of miR-514a-5p in NPC

**Fig. 3:**
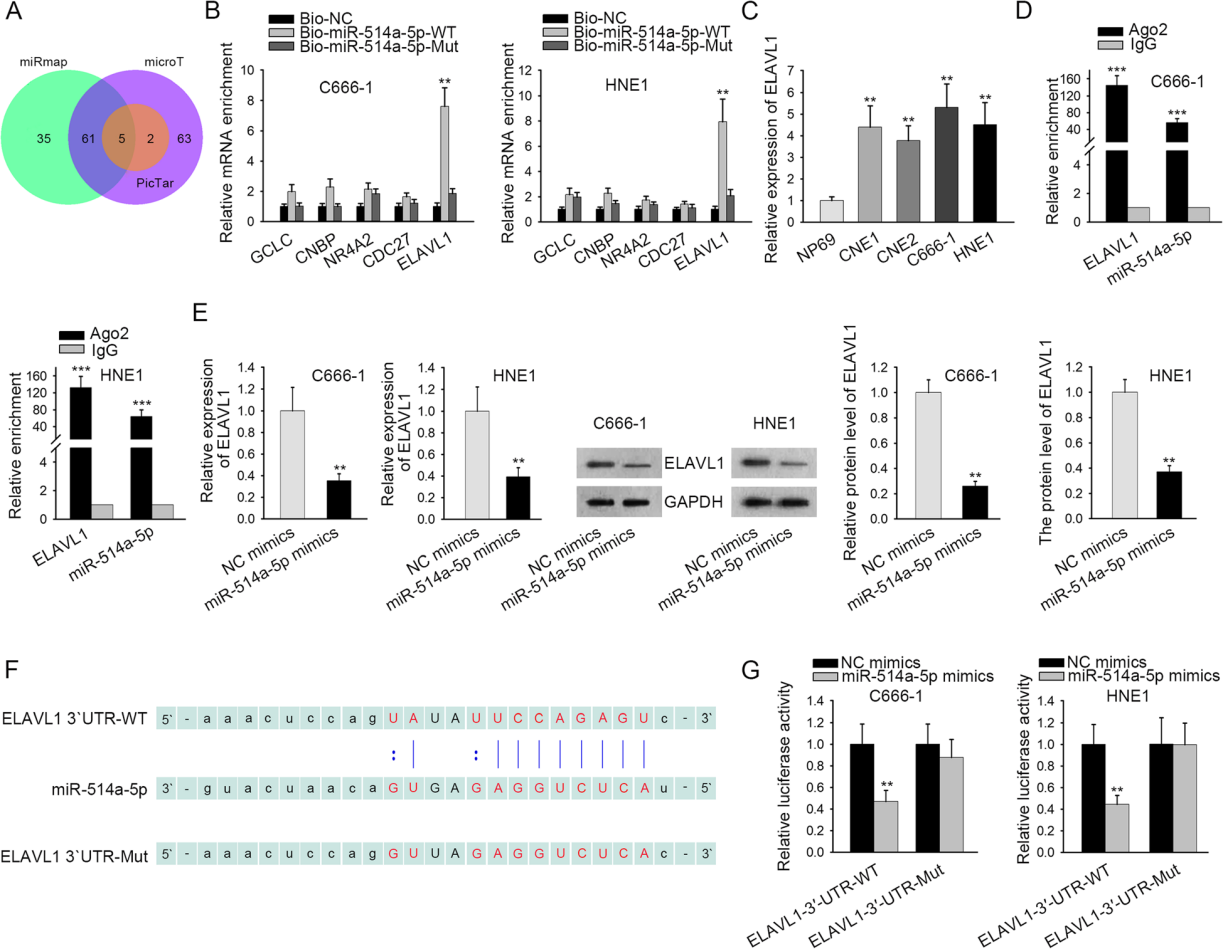
ELAVL1 is a downstream target gene of miR-514a-5p in NPC. **a** The downstream mRNAs of miR-514a-5p were obtained from starBase website. **b** RNA pull down assay explored the interaction between miR-514a-5p and its downstream mRNAs. **c** The expression of ELAVL1 in NPC cells and nasopharyngeal epithelial cell line was investigated by RT-qPCR. **d** It was manifested by RIP assay that ELAVL1 and miR-514a-5p coexisted in RISC. **e** The influence of miR-514a-5p overexpression on ELAVL1 mRNA and protein level was revealed by RT-qPCR and western blot assay respectively. These blots were cropped. The uncropped blots were shown in Supplementary figure 2. **f** The putative binding sites of ELAVL1 and miR-514a-5p, as well as the mutant sites in mutant-type ELAVL1 reporter were illustrated. **g** The binding ability between ELAVL1 and miR-514a-5p was proofed by luciferase reporter assay. GAPDH was used as an internal control. Error bars represent the mean ± SD of at least three independent experiments. ^**^P < 0.01, ^***^P < 0.001

Subsequently, starBase was utilized to find out the possible target genes of miR-514a-5p in NPC and 5 mRNAs (GCLC, CNBP, NR4A2, CDC27 and ELAVL1) were identified (Fig. 3a). RNA pull-down assay depicted that ELAVL1 was remarkably enriched in Bio-miR-514a-5p-WT rather than Bio-NC or Bio-miR-514a-5p-Mut group. Besides, no significant enrichments of other mRNAs were observed (Fig. 3b). Therefore, we conducted RT-qPCR to further measure the expression level of ELAVL1 in NPC cells. As shown in Fig. 3c, ELAVL1 expression was evidently upregulated in NPC cell lines compared with normal human nasopharyngeal epithelium. Further, RIP assay demonstrated that ELAVL1 and miR-514a-5p were enriched in anti-Ago2 group but not in anti-IgG group (Fig. 3d). Besides, the mRNA and protein expressions of ELAVL1 were reduced by miR-514a-5p overexpression in C666–1 and HNE1 cells (Fig. 3e and S2B). Moreover, the binding sites between ELAVL1 and miR-514a-5p were obtained from starBase (Fig. 3f). The luciferase activity of pmirGLO-ELAVL1-WT was reduced by miR-514a-5p overexpression whereas the luciferase activity of pmirGLO-ELAVL1-Mut showed no clear change under miR-514a-5p upregulation (Fig. 3g). Additionally, we confirmed that knockdown of SNHG7 reduced the mRNA and protein levels of ELAVL1 in two NPC cell lines (Figure S1B and S2D). All the findings above suggest that ELAVL1 is a downstream target gene of miR-514a-5p in NPC.

### SNHG7 promotes NPC progression via miR-514a-5p/ELAVL1 axis

**Fig. 4:**
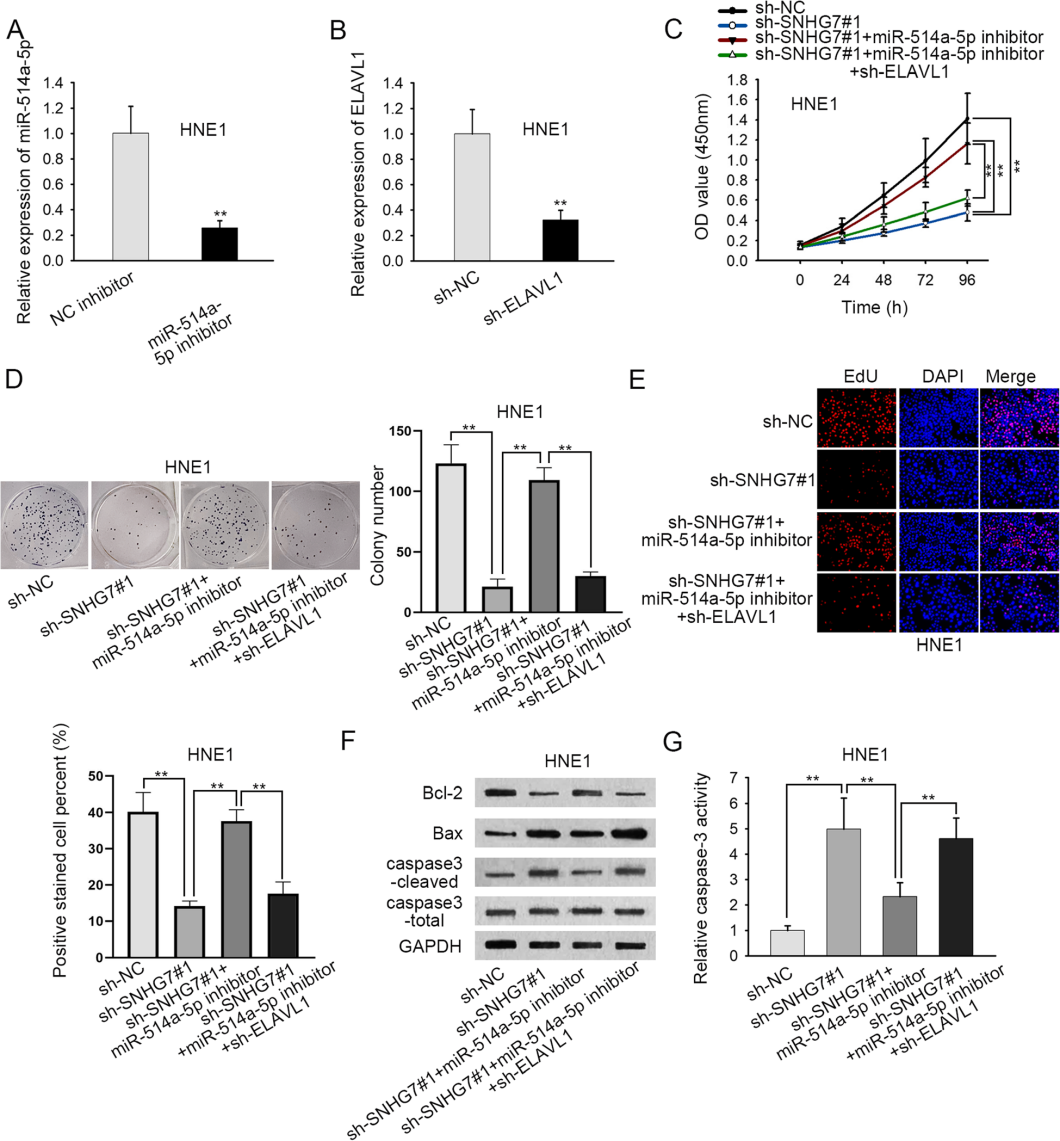
SNHG7 promotes NPC progression via miR-514a-5p/ELAVL1 axis. **a**-**b** The knockdown efficiency of miR-514a-5p and ELAVL1 was examined by RT-qPCR. **c**-**e** CCK-8, colony formation and EdU assays explored cell proliferation in different groups. **f** The levels of apoptosis-related proteins were identified by western blot assay. These blots were cropped. The uncropped blots were shown in Supplementary figure 2. **g** Cell apoptosis in different groups was revealed by caspase-3 activity. GAPDH was used as an internal control. Error bars represent the mean ± SD of at least three independent experiments. ^*^P < 0.05, ^**^P < 0.01

In order to further probe the regulatory mechanism of SNHG7 in NPC, rescue experiments were carried out after examining the knockdown efficiency of miR-514a-5p or ELAVL1. As depicted in Fig. 4a-b, the expression of miR-514a-5p was decreased by miR-514a-5p inhibitor and the expression of ELAVL1 was reduced by sh-ELAVL1 in HNE1 cells. According to CCK-8, colony formation as well as EdU assays, cell proliferation was increased in sh-SNHG7#1 + miR-514a-5p inhibitor group compared with sh-SNHG7#1 group, and such increase was counteracted by the co-transfection of sh-ELAVL1 (Fig. 4c-e). Next, western blot assay uncovered that miR-514a-5p inhibitor reversed the low expression of Bcl-2 and high expression of Bax and caspase 3-cleaved caused by sh-SNHG7#1, and sh-ELAV1 counteracted the effect of miR-514a-5p mentioned above (Fig. 4f and S2C). Finally, HNE-1 cells presented a decrease of caspase-3 activity in sh-SNHG7#1 + miR-514a-5p inhibitor group compared with sh-SNHG7#1 group, and such decrease was reversed by sh-ELAVL1 (Fig. 4g). To sum up, SNHG7 promotes NPC progression by targeting miR-514a-5p/ELAVL1 axis.

## Discussion

Recently, a series of researches have indicated that lncRNAs could function as ceRNAs to regulate the progression of human cancers [23], such as oral squamous cell carcinoma [24], lung cancer [25], colorectal cancer [26], and so on. It has been reported that lncRNA SNHG7 promotes cell proliferation, migration and invasion in pancreatic cancer by sponging miR-342-3p and targeting ID4 [27]. LncRNA SNHG7 is associated with tumor stage and lymph node metastasis of breast cancer via regulating miR-186 [28]. LncRNA SNHG7 facilitates the proliferation and cycle progression of prostate cancer through miR-503/cyclin D1 axis [29]. A recent study showed that SNHG7 was upregulated in NPC tissues and that knockdown of SNHG7 inhibited proliferation, migration, and invasion of NPC cells via downregulating ROCK1 [21], but the mechanism of SNHG7 still needs to be further explained. In consistence, our data also validated that the relative expression of SNHG7 was significantly upregulated in NPC cells, and SNHG7 facilitated the proliferation whereas repressed the apoptosis of NPC. All the data suggested that lncRNA SNHG7 played an oncogenic role in NPC.

MicroRNAs (miRNAs) are single-stranded transcripts consisting of around 25 nucleotides, and cannot encode the proteins [30]. Our data confirmed the cytoplasmic distribution of SNHG7 in NPC cells. Previous studies have confirmed that cytoplasmic lncRNAs can interact with specific miRNAs to regulate the development of human cancers [31]. For example, lncRNA TUG1 regulates the proliferation and apoptosis of osteosarcoma by sponging miR-212-3p [32]. LncRNA HOTAIR promotes progression of colorectal cancer by sponging miR-197 [33]. LncRNA PVT1 accelerates the oncogenesis of non-small-cell lung cancer through regulating miR-497 [34]. LncRNA SNHG7 has been explored to promote the tumor growth and EMT process in osteosarcoma via regulating miR-34a [35]. Our study was the first to reveal that SNHG7 could combine with miR-514a-5p in NPC cells. In a word, lncRNA SNHG7 acted as a sponge for miR-514a-5p.

ELAVL1 has been reported as an oncogene in many cancers and closely associated with tumor progression [36–38]. It was widely identified that miRNAs could inhibit the translation of mRNAs to decrease tumorigenesis through binding to specific target mRNAs [39]. MiR-324-5p suppresses cell growth and invasion by regulating ELAVL1 in colorectal cancer [40]. MiRNA-9 inhibits the pyroptosis induced by hyperglycemia through targeting ELAVL1 in human ventricular cardiomyocytes [41]. Moreover, lncRNA MALAT1 modulates renal tubular epithelial pyroptosis in diabetic nephropathy via sponging miR-23c and targeting ELAVL1 [42]. In the present study, we firstly verified that ELAVL1 was a downstream gene of miR-514a-5p, and the expression level of ELAVL1 was negatively regulated by miR-514a-5p and positively regulated by SNHG7. In addition, miR-514a-5p inhibitor could partially counteract promoting effect of SNHG7 knockdown on NPC progression, and ELAVL1 silence reserved the effect of sh-SNHG7 + miR-514a-5p inhibitor on NPC progression.

## Conclusion

To be concluded, lncRNA SNHG7 promotes the proliferation and migration of NPC by miR-514a-5p/ELAVL1 axis, indicating that SNHG7/miR-514a-5p/ELAVL1 axis could serves as an underlying diagnostic/therapeutic target for NPC.

## Supplementary information

**Additional file 1: Figure S1.** (A) quantification of western blot results of Fig. 1f. (B) qRT-PCR and western blot analysis of ELAVL1 level in C666–1 and HNE-1 cells under sh-SNHG7#1 compared with sh-NC. These blots were cropped. The uncropped blots were shown in Supplementary figure 2. ^**^P < 0.01.

**Additional file 2: Figure S2.** Original protein bands of Figs. 1f, 3e, 4f and S1B.

## Data Availability

Not applicable.

## Abbreviations

Ago2: Argonaute-2
ANOVA: Analysis of variance
CCK-8: Cell counting kit-8
cDNA: complementary deoxyribose nucleic acid
ceRNA: competing endogenous RNA
CO2: Carbon dioxide
ECL: Enhanced chemiluminescence
GAPDH: Glyceraldehyde-3-phosphate dehydrogenase
IgG: Immunoglobulin G
lncRNA: long noncoding RNA
miRNA: microRNA
NPC: Nasopharyngeal carcinoma
PBS: Phosphate-buffered saline
PVDF: Polyvinylidene fluoride
RIPA: Radio Immunoprecipitation Assay
RISC: RNA induced silencing complex
RT-qPCR: Real-time reverse-transcription polymerase chain reaction
SD: Standard deviation
SDS-PAGE: Sodium dodecyl sulfate polyacrylamide gel electrophoresis
shRNA: short hairpin RNA
SPSS: Statistical Product and Service Solutions
