# Supplementary figures and images for: LncRNA SNHG7 promotes the proliferation of nasopharyngeal carcinoma by miR-514a-5p/ELAVL1 axis
Source: BMC Cancer. 2020 May 5;20:376. doi: 10.1186/s12885-020-06775-8 (PMC7202000; doi:10.1186/s12885-020-06775-8)

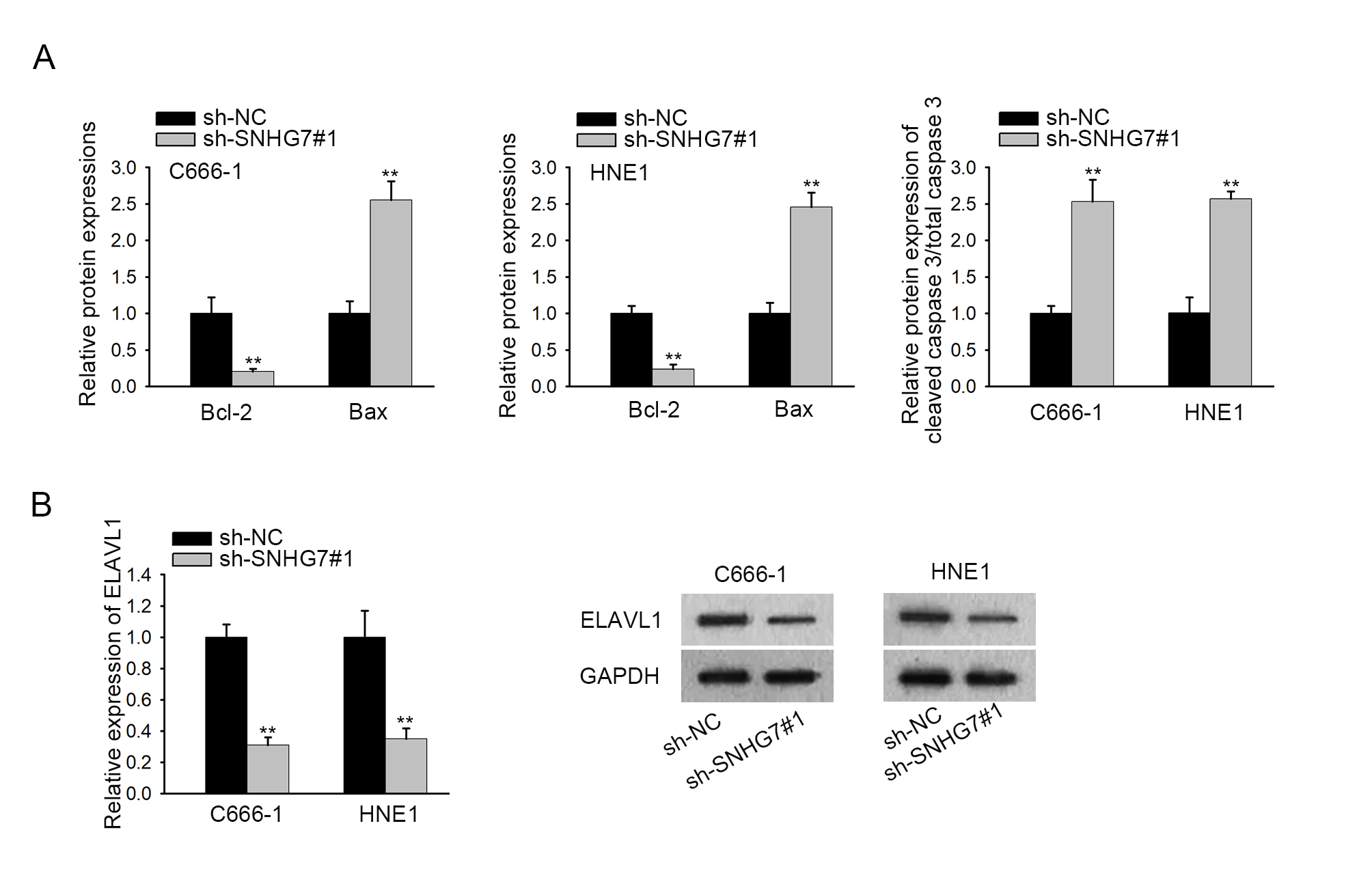

Supplement: Supplementary file 1 — Additional file 1: Figure S1. (A) quantification of western blot results of Fig. 1f. (B) qRT-PCR and western blot analysis of ELAVL1 level in C666–1 and HNE-1 cells under sh-SNHG7#1 compared with sh-NC. These blots were cropped. The uncropped blots were shown in Supplementary figure 2. **P < 0.01. [file 12885_2020_6775_MOESM1_ESM.tif]

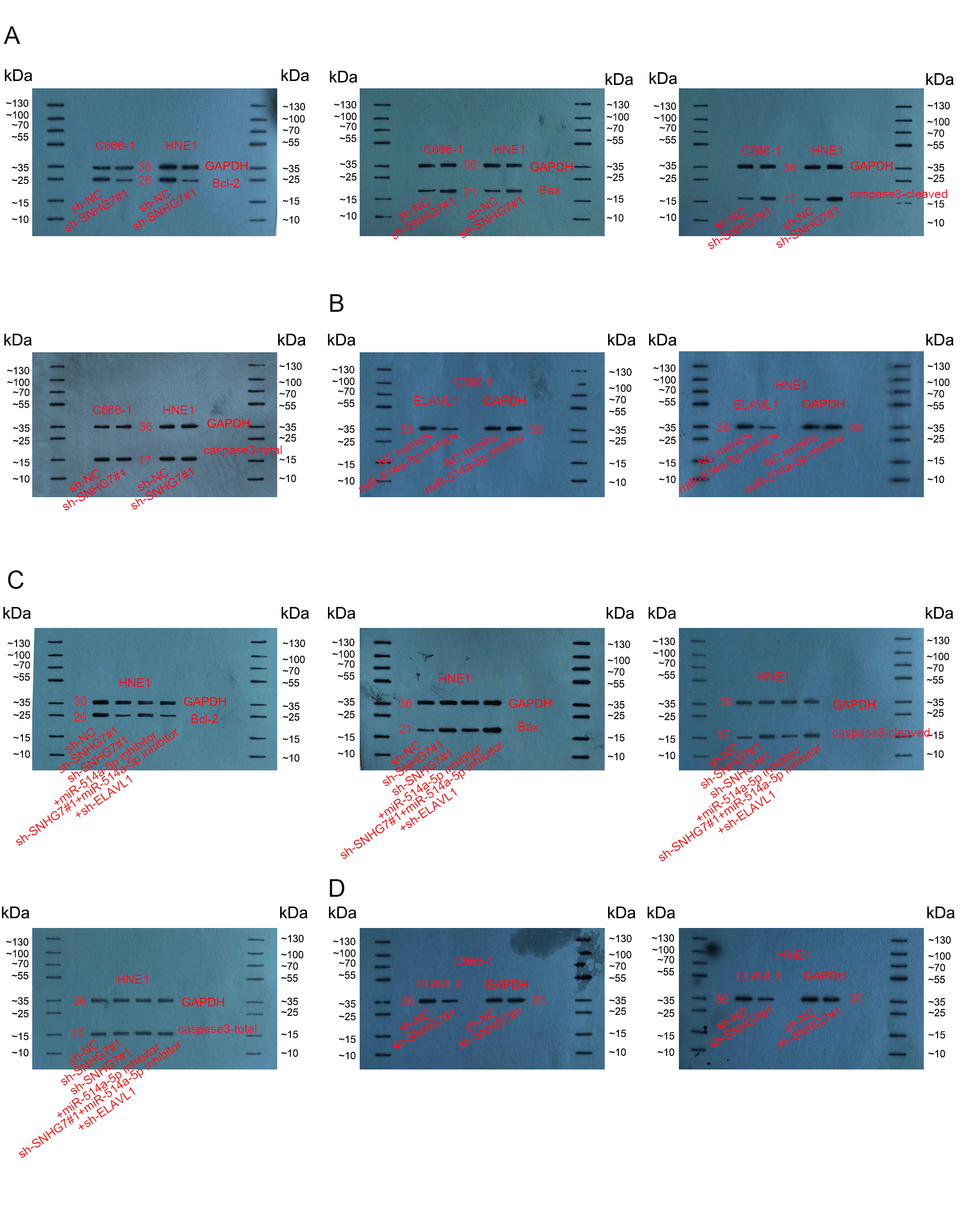

Supplement: Supplementary file 2 — Additional file 2: Figure S2. Original protein bands of Figs. 1f, 3e, 4f and S1B. [file 12885_2020_6775_MOESM2_ESM.tif]
